# A Case Report of a Strangulated Diaphragmatic Laceration: An Uncommon Late Complication of Cardiac Ablation

**DOI:** 10.3390/reports8020048

**Published:** 2025-04-13

**Authors:** Luca Ghirardelli, Luana Genova, Giuseppe D’Angelo, Caterina Bisceglia, Michele Carlucci

**Affiliations:** 1 Chirurgia Generale e delle Urgenze, IRCCS San Raffaele Scientific Institute, 20132 Milan, Italy; 2 Unità Operativa di Aritmologia ed Elettrofisiologia Cardiaca, IRCCS San Raffaele Scientific Institute, 20132 Milan, Italy

**Keywords:** ventricular ablation, endocardial ablation, case report, diaphragmatic hernia, laparoscopy treatment of diaphragmatic hernia

## Abstract

**Background and Clinical Significance:** In recent years, the catheter ablation of cardiac arrhythmias has significantly reduced the incidence of sudden cardiac deaths and the need for chronic antiarrhythmic therapy. Endocardial ablation of ventricular arrhythmias is less common than atrial ablation and is technically more challenging. There are few documented extracardiac complications for ventricular ablation, and there is no report of diaphragmatic laceration. **Case Presentation:** We report a case of acute diaphragmatic laceration following endovascular ventricular ablation resulting in the strangulation of the gastric fundus in a patient who experienced previous transcutaneous ventricular ablation two years before. The patient underwent exploratory laparoscopy, revealing a diaphragmatic laceration with incarceration of the gastric fundus. Resection of the gastric fundus, showing acute ischemic damage, and closure of the diaphragmatic defect near the right ventricle with sutures were required. No complications were observed in the postoperative course. **Conclusions:** Although diaphragmatic injury is extremely rare, it should be considered among the complications associated with ventricular ablation.

## 1. Introduction and Clinical Significance

Sudden cardiac death (SCD) remains a major public health concern, with recent estimates indicating an annual incidence of 39.7 cases per 100,000 individuals in Europe [1]. A significant proportion of these deaths is attributable to ventricular arrhythmias. Since the mid-20th century, the introduction of defibrillation as a treatment for ventricular arrhythmias has meant that such events are no longer necessarily fatal. However, while implantable cardioverter–defibrillators (ICDs) are life-saving, they do not prevent ventricular arrhythmias and may negatively impact quality of life in patients experiencing frequent appropriate shocks. Percutaneous ablation of ventricular tachycardia (VT) is an effective therapeutic strategy for eliminating VT and preventing its recurrence. There are two percutaneous approaches: the endocardial and the endo-epicardial. In the first, radiofrequency is applied exclusively from the endocardial side, while in the second, an additional ablation catheter is used, inserted through the thoracic wall, usually in the subxiphoid region. The most common complications associated with endocardial VT ablation relate to vascular access. Less common complications include acute hemodynamic decompensation, cardioembolic events, and those related to catheter manipulation, such as valvular injury, aortic dissection, dislodgement of cardiac implantable electronic devices, pro-arrhythmia related to ablation, and collateral damage resulting from ablation, including conduction system injury, coronary artery injury, and steam pops [2]. The reported endo-epicardial VT ablation complication rate ranges from 1% to 14%. The most common complications include right ventricular puncture (5–14%), acute hemopericardium (4–11%), and acute tamponade (5%). Less frequent complications include coronary occlusion (0.9%), phrenic nerve paralysis (0.6%), and hepatic or subxiphoid hematomas (0.5%) [3,4,5,6]. No diaphragmatic laceration and subsequent gastric strangulation have been reported in the current literature.

## 2. Case Presentation

### 2.1. Patient Information and Symptoms

We report a case of a 77-year-old man with dilated cardiomyopathy related to a previous myocarditis. He presented with multiple ICD shocks for recurrent ventricular tachycardia. The patient was treated in 2017 in a different center, with endo-epicardial VT ablation of two arrhythmogenic foci (inferior and ifero-lateral) using a subxiphoid approach. No adverse events were registered after the procedure. Redo endocardial procedure was indicated because of arrhythmia recurrence. The patient came to the attention of the emergency surgeon after experiencing multiple episodes of hematemesis during hospitalization in the cardiovascular department.

### 2.2. Diagnosis and Preoperative Evaluation

**Figure 1:**
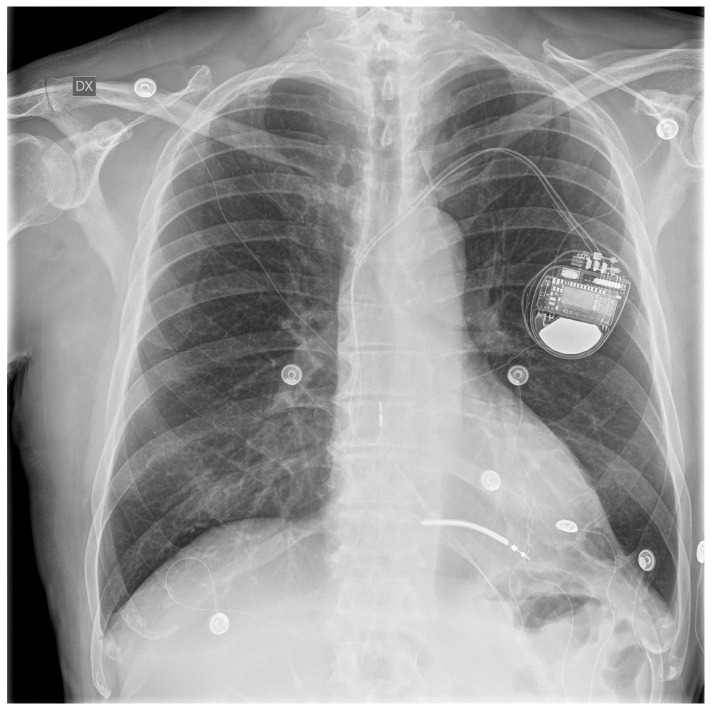
Preoperative chest X-ray.

**Figure 2:**
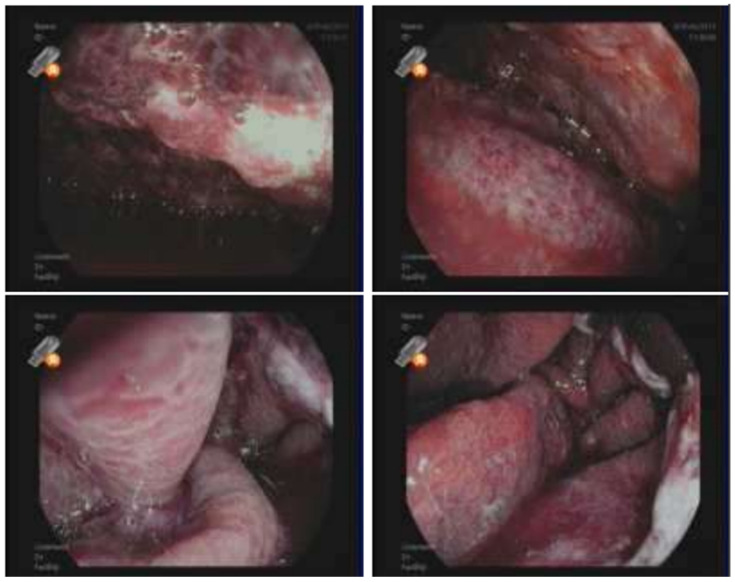
Upper GI endoscopy showing gastric ischemic mucosae.

Before completing the preprocedural assessment, including normal chest X-ray (Figure 1), the patient underwent transcatheter ablation of ventricular tachycardia with an endocardial approach. At the end of the procedure, the patient exhibited multiple episodes of vomiting and coughing in the recovery room. In the next 48 h, multiple episodes of hematemesis occurred; the hemoglobin level was 12.5 g/dL (12–16 g/dL), and the patient had no signs of hemodynamic impairment. Physical examination revealed only mild epigastric pain. Upper GI endoscopy evaluation was urgently performed, revealing gastric mucosa with a diffusely hemorrhagic appearance and grayish-colored ischemic mucosa (Figure 2).

**Figure 3:**
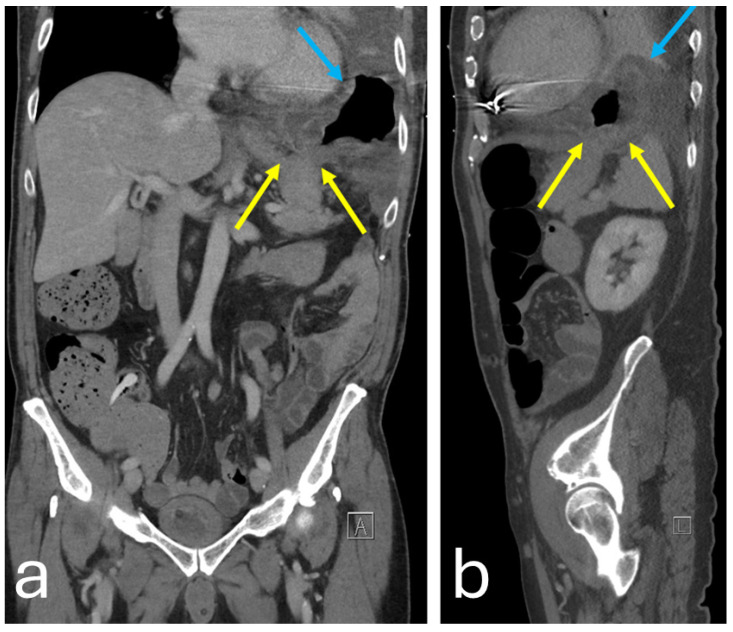
CT scan of coronal (**a**) and sagittal (**b**) planes showing diaphragmatic laceration (yellow arrows) with incarcerated gastric fundus (blue arrows).

Chest–abdominal contrast-enhanced computer tomography (CT) demonstrated herniation of the gastric fundus into the thorax through discontinuation of the left diaphragmatic dome. The mucosa of the gastric fundus appeared thickened due to edema and delayed arterial enhancement compared with the remaining portions of the stomach, suggesting possible ischemic suffering (Figure 3).

Concomitant left pleural effusion with a maximum thickness of approximately 4.5 cm was associated with almost complete atelectasis of the ipsilateral lobe. After completing the preoperative assessment and anesthesia evaluation, the patient underwent diagnostic laparoscopic surgery with four trocar techniques.

### 2.3. Surgical Intervention

**Figure 4:**
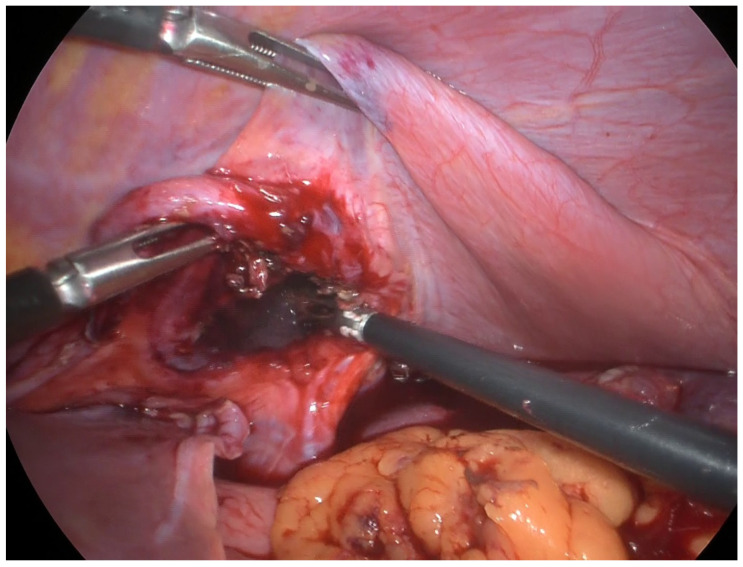
Diaphragmatic laceration after gastric reduction.

During the operation, thoracic stomach herniation through the left anterior diaphragmatic discontinuity was confirmed. The diaphragmatic laceration was close to the right ventricle. It was necessary to enlarge the diaphragmatic discontinuity (maximum width 5 cm) to reduce the body and fundus of the stomach that showed a transmural hematoma. There was no evidence of a hernia sac during surgery, confirming the hypothesis of a post-cardiac procedure laceration of the diaphragm despite a diagrammatical hernia (Figure 4).

Gastric resection was performed using the mechanical stapler, Echelon^TM^ (Johnson&Johnson, New Brunswick, NJ, USA), due to ischemic tissue damage. The diaphragmatic defect was sutured using Ti-cronTM (Covidien, Dublin, Ireland) and PTFE pledget (Teleflex Medical, Wayne, PA, USA).

### 2.4. Postoperative Course and Follow-Up

On the fifth postoperative day, the patient underwent a radiographic study of the transit with a contrast medium taken orally (Gastrografin^®^), which was negative for leakage. The patient began a well-tolerated soft and fractionated diet, which was upgraded to a free diet. After three years, the patients underwent a CT showing no signs of hernial recurrence.

## 3. Discussion

Epicardial approach is frequently indicated in VT ablation, especially in non-ischemic etiologies. Complications related to the epicardial approach range from 1% to 14% in the current literature [7]. These include phrenic nerve injuries and hemoperitoneum secondary to diaphragmatic, left hepatic lobe, or internal mammary artery injury [8]. Subdiaphragmatic hematomas are reported only in the inferior approach for epicardial access because the needle frequently crosses the diaphragmatic dome. In our case, various clues led us to believe that the epicardial procedure performed a few years prior may have caused a weakening of the diaphragm, which is attributable to the mechanical and thermal damage from the radiofrequency catheter, although without a definitive diagnosis (e.g., histological). The mechanical injury, related to the introduction of the catheter through the diaphragmatic tissue (often repeated during this procedure), may have caused penetrating microlesions that subsequently evolved into an area of weakness. In addition, the thermal injury may have led to muscle damage with subsequent progression to a fibrotic area, characterized by reduced elasticity.

Moreover, the cardiac regions subjected to ablation (and thus to direct thermal injury) were the inferior and inferolateral areas of the right ventricle, which are anatomically in direct contact with the diaphragmatic dome. We believe that the conjunction of diaphragmatic weakness and increased abdominal pressure due to vomiting could be implicated in the diaphragmatic injury and subsequent gastric incarceration. The absence of a hernia sac indicated a diaphragmatic laceration rather than a primary hernia. The preprocedural chest X-ray did not document gastric herniations, suggesting the acute onset of diaphragmatic injury (approximately 2 days after the endocardial procedure). Furthermore, we did not identify any additional predisposing factors for diaphragmatic laceration in the patient, including connective tissue disorders, previous surgeries, or abdominal trauma.

The symptoms of complicated diaphragmatic hernias can vary widely, ranging from obstructive symptoms, if stomach, small intestine, or colon herniation occurs, to respiratory symptoms secondary to pleural cavity obstruction by herniated tissue/organ. Sometimes, the hernia may remain entirely asymptomatic at initial presentation or even for several years, with only nonspecific symptoms developing over time until incarceration or strangulation becomes a surgical emergency. In these cases, the herniated tissue undergoes ischemic damage, and occlusive symptoms are compounded by the Systemic Inflammatory Response Syndrome (SIRS) related to tissue damage, with a mortality rate reaching 66% [9]. Unfortunately, pain often manifests late when ischemic damage is too advanced. The first symptom manifested by the patient (on anticoagulant therapy) was hematemesis without pain in a hemodynamically stable patient, so an upper GI endoscopy was performed, unexpectedly revealing ischemia of the gastric mucosa and necessitating a chest–abdomen CT scan.

This examination has a high specificity (87%) in identifying complicated diaphragmatic hernias, while sensitivity decreases significantly in cases of hernia without incarceration (82% vs. 14%) [10]. However, the CT scan is considered the diagnostic gold standard. Various CT signs have been described for traumatic diaphragmatic hernias (TDHs) that can also be used in this type of lesion. While diagnostic accuracy increases with the presence of multiple signs, even a single sign should raise suspicion for TDHs. CT signs have been identified and categorized into three groups: direct signs, indirect signs, and those of uncertain or controversial origin. Direct signs include an absent diaphragm, segmental diaphragmatic defects, and the Dangling Diaphragm sign. Indirect signs are further divided into those related to herniation and those related to a loss of the border between the thorax and abdomen. Herniation-related signs include herniation through a defect, the Collar sign (also known as the hourglass constriction sign or mushroom sign in hepatic hernia), the hump sign, the band sign, dependent viscera, the sinus cut-off sign, abdominal contents positioned peripherally to the diaphragm or lung, and elevated abdominal organs. Border-loss signs include abdominal fluid in direct contact with thoracic structures, abdominal viscera adjacent to thoracic fluid or organs, pneumothorax/pneumoperitoneum, and hemothorax/hemoperitoneum. Signs of uncertain or controversial origin include diaphragmatic thickening (curled diaphragm sign), contrast-medium extravasation in the diaphragmatic or peridiaphragmatic region, and a hypoenhanced or hypoattenuated diaphragm. The sensitivity of CT in detecting diaphragmatic scarring or weakening after thermal and mechanical insults, such as ablation, is not well documented in the literature. For this reason, in our opinion, the routine use of this imaging modality in patients undergoing epicardial ablation to identify potential diaphragmatic weakening is not justified. Although no specific data are available in the literature, a simple preoperative X-ray could already reveal pre-existing lacerations or herniations and is often included in the standard preprocedural evaluation of these patients.

Surgery is the treatment of choice for complicated diaphragmatic hernias, as in this case [11]. The approach can be either laparoscopic or thoracoscopic. Recently, the thoracoscopic and laparoscopic approach has become more widely accepted in treating these types of lesions, although in emergencies, the laparotomy technique is more commonly used. The diagnostic and therapeutic value of laparoscopy and thoracoscopy is indispensable in situations where the diagnosis often remains unconfirmed through imaging. Patients experience the benefits of minimally invasive surgery, such as reduced postoperative pain, faster recovery, and improved cosmetic outcomes [12,13]. The standard approach for addressing traumatic diaphragmatic hernias (TDHs) involves reducing abdominal viscera into the abdomen and subsequently repairing the diaphragmatic defect. This repair can be carried out through either a thoracic or abdominal approach. Thoracic surgeons typically prefer the thoracic approach, while general surgeons commonly opt for the abdominal approach. An abdominal approach becomes necessary when there is suspicion of bowel ischemia and necrosis or other traumatic injuries to the abdominal viscera. Debates revolve around the type of closure. Most defects, especially smaller ones, can be repaired primarily. The American Association for the Surgery of Trauma (AAST) classifies diaphragmatic injuries into five grades: Grade I (contusion), Grade II (laceration ≤ 2 cm), Grade III (laceration 2–10 cm), Grade IV (laceration > 10 cm with tissue loss ≤ 25 cm^2^), and Grade V (laceration with tissue loss > 25 cm^2^) [14]. Extensive defects, typically those classified above Grade III, often require reinforcement with a mesh. This can be secured using sutures, tacks, or cyanoacrylate glue [15]. In such instances, achieving a tension-free repair involves the use of a prosthetic material to compensate for tissue loss and ensure structural stability. Various prosthetic options are available, including a standard polypropylene mesh, composite mesh, polytetrafluoroethylene (PTFE) mesh, and biological mesh. In our case, we managed an AAST Grade III injury and proceeded with primary defect repair following the repositioning of the herniated contents. In this patient, despite the anatomical proximity of the pericardium and the right ventricle, no special precautions were necessary. The diaphragm, being a thick and fibrous muscle, allows for the application of sutures with a negligible risk of cardiac injury.

To date, the patient has not exhibited any symptoms suggestive of recurrence. In the literature, data on the recurrence rate after direct suturing of a diaphragmatic laceration are limited and often based on inadequate follow-up [16].

## 4. Conclusions

In conclusion, the presented case represents a possible late complication not reported in the literature after ventricular epicardial ablation. The uniqueness of this case report does not allow for the identification of specific risk factors for this occurrence. However, it is important to note that the epicardial approach may increase the risk of extracardiac abdominal complications, especially when lower access is used.

In patients undergoing this procedure, the onset of unusual symptoms such as abdominal pain, vomiting, dysphagia, or hematemesis should prompt clinicians to consider this potential complication. The difficulty lies in the early identification of the problem to limit organ damage and the subsequent increase in postoperative morbidity and mortality. The diagnostic and therapeutic work-up follows that of post-traumatic diaphragmatic hernias.

## Data Availability

The original contributions presented in this study are included in the article. Further inquiries can be directed to the corresponding author.

## Abbreviations

The following abbreviations are used in this manuscript:

SCD	Sudden cardiac death
ICDs	Implantable cardioverter–defibrillators
VT	Ventricular tachycardia
CT	Contrast-enhanced computer tomography
SIRS	Systemic Inflammatory Response Syndrome
TDHs	Traumatic diaphragmatic hernias
AAST	American Association for the Surgery of Trauma
PTFE	Polytetrafluoroethylene
